# Predictive Value of the Estimated Dose of Radiation to Immune Cells Versus Conventional Parameters in Elderly Patients With Unresectable Stage III NSCLC: A Two‐Center Real‐World Study

**DOI:** 10.1111/1759-7714.70196

**Published:** 2025-12-17

**Authors:** Huan Li, Xingyu Du, Song Guan, Hui Wang, Yan Xing, Cuimeng Tian, Li Wen

**Affiliations:** ^1^ Department of Emergency, Beijing Chest Hospital Capital Medical University & Beijing Tuberculosis and Thoracic Tumor Research Institute Beijing China; ^2^ Department of Radiation Oncology, Beijing Chest Hospital Capital Medical University & Beijing Tuberculosis and Thoracic Tumor Research Institute Beijing China; ^3^ Department of Respiratory and Critical Care Hebei PetroChina Central Hospital Langfang Hebei China

**Keywords:** dosimetric parameters, EDRIC, non‐small cell lung cancer, predictive value, radiotherapy

## Abstract

**Objective:**

To compare the predictive value of the estimated dose of radiation to immune cells (EDRIC) with conventional dosimetric parameters for survival in elderly patients with stage III unresectable NSCLC after chemoimmunotherapy and radiotherapy.

**Methods:**

We conducted a retrospective study of elderly patients (≥ 65 years) treated at two institutions. Patients were stratified by median EDRIC, mean lung dose (MLD), mean heart dose (MHD), and mean body dose (MBD). Survival was analyzed using Kaplan–Meier, Cox regression, and ROC curves.

**Results:**

Baseline characteristics were well‐balanced across dosimetric parameter subgroups (all *p* > 0.05). The median progression‐free survival (PFS) and overall survival (OS) for the entire cohort were 23.9 months and 46.0 months, respectively. EDRIC ≥ 6.4 Gy was associated with worse PFS (*p* = 0.019) and OS (*p* = 0.011), while MLD, MHD, and MBD showed no prognostic significance (all *p* > 0.05). Multivariate analysis identified EDRIC ≥ 6.4 Gy as an independent predictor of worse PFS (HR = 1.852, *p* = 0.049) and OS (HR = 2.289, *p* = 0.048). Age ≥ 70 years was also independently associated with poorer OS (HR = 2.870, *p* = 0.011). ROC analysis demonstrated superior predictive performance of EDRIC over conventional parameters for 1‐, 2‐, and 3‐year PFS and OS, with particularly outstanding discrimination for 12‐month OS (AUC = 0.93).

**Conclusion:**

EDRIC shows potential in predicting survival for elderly stage III unresectable NSCLC patients, with 6.4 Gy as a potential threshold for personalized radiotherapy optimization. These findings require prospective validation.

## Graphical abstract

In elderly patients with unresectable stage III NSCLC receiving radiotherapy after chemoimmunotherapy, an EDRIC ≥ 6.4 Gy independently predicted poorer survival. EDRIC outperformed conventional dosimetric parameters, suggesting its value in personalizing radiotherapy to improve outcomes.

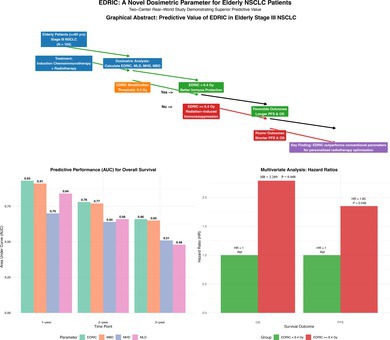

## 1 Introduction

Elderly patients (≥ 65 years) with unresectable stage III non‐small cell lung cancer (NSCLC) represent a clinically challenging subgroup due to tumor invasiveness, comorbidities, and physiological decline, which often precludes surgical intervention [1]. This population faces a therapeutic dilemma: diminished treatment tolerance due to immunosenescence and reduced organ reserve function leads to poorer outcomes compared to younger patients. Specifically, elderly patients exhibit lower completion rates of concurrent chemoradiotherapy (cCRT) (47% vs. 57%), higher rates of severe toxicity (19.2% vs. 7.6%), and a 5‐year overall survival (OS) deficit of 10%–15% [2, 3]. While immune checkpoint inhibitor (ICI) consolidation therapy has improved prognosis in broader NSCLC populations, its benefits in elderly subgroups remain uncertain, highlighting the need for more precise therapeutic strategies [4, 5].

Induction chemoimmunotherapy has demonstrated survival advantages in non‐elderly NSCLC patients, with a reported median OS of 27.9 months compared to 20.2 months with chemotherapy [6]. However, elderly patients exhibit greater heterogeneity in treatment response and experience higher rates of immune‐related adverse events (irAEs), raising concerns about the risk–benefit balance in this population [4, 5, 7, 8]. Age‐related immune dysfunction—including T‐cell exhaustion, altered tumor‐infiltrating lymphocyte (TIL) activity, and impaired STING pathway activation—may further diminish the predictive utility of conventional biomarkers such as PD‐L1 expression [5, 7, 9]. Consequently, novel assessment tools tailored to the immunosenescent microenvironment are urgently needed.

The estimated dose of radiation to immune cells (EDRIC) quantifies radiation‐induced immunosuppression by assessing cumulative damage to immune organs (e.g., thymus, lymph nodes). In NSCLC patients receiving postoperative radiotherapy, elevated EDRIC correlates with worse OS and progression‐free survival (PFS), mediated by radiation‐induced suppression of TILs and enrichment of myeloid‐derived suppressor cells (MDSCs) [10, 11, 12]. Notably, elderly patients exhibit unique immunosenescent features, including expanded MDSC populations and impaired STING pathway activation, which may exacerbate radiotherapy‐induced immunosuppression and alter EDRIC's prognostic implications [13]. Although EDRIC's association with poor outcomes has been validated in some cohorts, its predictive superiority over conventional dosimetric parameters in elderly NSCLC patients remains unverified [14, 15]. Addressing this gap is critical to developing personalized treatment strategies for this vulnerable population.

To this end, we conducted a two‐center real‐world study to evaluate EDRIC's predictive value versus traditional dosimetric parameters in elderly patients with unresectable stage III NSCLC.

## 2 Materials and Methods

### 2.1 Study Population

This retrospective, two‐center study included patients diagnosed with unresectable stage III NSCLC from Beijing Chest Hospital, Capital Medical University, and Hebei PetroChina Central Hospital between January 2014 and June 2024. Inclusion criteria: (1) Histologically or cytologically confirmed stage III unresectable NSCLC (based on the eighth edition of the AJCC staging system); (2) Aged ≥ 65 years; (3) Received radical radiotherapy sequentially or synchronously after immunochemotherapy induction containing PD‐1/PD‐L1 inhibitors; (4) Complete clinical and dosimetric data. Exclusion criteria: (1) Previous receipt of targeted therapy or other tumor‐specific treatments; (2) No radiotherapy received or only immune monotherapy; (3) Missing key clinical data.

This study was approved by the Ethics Committee of Beijing Chest Hospital, Capital Medical University, in line with the *Declaration of Helsinki*, and all patients signed informed consent forms.

### 2.2 Data Collection and Grouping

The following information was collected through the electronic medical record system: (1) Demographic characteristics: age, gender, smoking history; (2) Clinicopathological characteristics: ECOG performance status score, pathological type, TNM stage; (3) Treatment details: chemoradiotherapy mode, radiotherapy dose; (4) Dosimetric parameters: mean lung dose (MLD), mean heart dose (MHD), mean body dose (MBD), and EDRIC.

Patients were grouped based on the median values of the above indicators: EDRIC < 6.4 Gy group versus ≥ 6.4 Gy group, MLD < 12 Gy group versus ≥ 12 Gy group, MHD < 10 Gy group versus ≥ 10 Gy group, MBD < 6 Gy group versus ≥ 6 Gy group, for subsequent analysis and comparison.

### 2.3 EDRIC Calculation

Dosimetric data are extracted from the radiotherapy planning system. The specific calculation formula for EDRIC is as follows:

$$\begin{array}{c} \text{EDRIC}=0.12\times \mathrm{MLD}+0.08\times \mathrm{MHD}\\ \;+\left[0.45+0.35\times 0.85\times {\left(\frac{\#\text{of fractions}}{45}\right)}^{\frac{1}{2}}\right]\times \mathrm{MBD} \end{array}$$

### 2.4 Treatment

#### 2.4.1 Immunochemotherapy Induction

Patients received platinum‐containing doublet chemotherapy combined with PD‐1/PD‐L1 inhibitors (camrelizumab, nivolumab, pembrolizumab, sintilimab, or tislelizumab). Chemotherapy regimens were selected according to pathological types: for squamous cell carcinoma, gemcitabine/paclitaxel + cisplatin/carboplatin was used; for non‐squamous cell carcinoma, pemetrexed + cisplatin/carboplatin was used.

#### 2.4.2 Radiotherapy Regimen

Intensity‐modulated radiotherapy (IMRT) was adopted, with positioning CT covering the neck, chest, and upper abdomen. Target volume definition: gross tumor volume (GTV) included the primary lesion and metastatic lymph nodes; clinical target volume (CTV) included GTV and potential micrometastatic areas; planning target volume (PTV) was CTV plus margins for movement and setup errors. The median prescribed dose was 60 Gy (range 40–66 Gy) with conventional fractionation (1.8–2.0 Gy per fraction).

### 2.5 Outcomes and Follow‐Up

The primary endpoints of this study were PFS and OS. According to the RECIST 1.1 criteria, PFS was the time from the start of immunochemotherapy induction to disease progression or death; OS was the time from the start of immunochemotherapy induction to death from any cause. In addition, receiver operating characteristic curves were used to evaluate the predictive efficacy of 1‐, 2‐, and 3‐year PFS rates and OS.

All patients received systematic follow‐up, including outpatient re‐examinations and telephone follow‐ups. Efficacy evaluation was performed every two treatment cycles during treatment. After the end of treatment, chest CT examinations were performed every 3 months in the first 2 years, and then follow‐up was conducted every 6 months until disease progression or patient death.

### 2.6 Statistical Analysis

R 4.0.3 software was used for analysis. The χ^2^ test or Fisher's exact test was used for comparison of baseline characteristics between groups. Survival analysis was performed using the Kaplan–Meier method to draw curves, and the log‐rank test was used to compare differences between groups; univariate and multivariate Cox proportional hazards regression models were used to calculate HR and 95% CI to evaluate the association between indicators and PFS, OS. Patients were stratified into high and low groups based on the median values of the dosimetric parameters (MLD: 12 Gy; MHD: 10 Gy; MBD: 6 Gy; EDRIC: 6.4 Gy) for subsequent survival analysis and comparison. The AUC value was calculated through the ROC curve to compare the predictive accuracy. A two‐tailed *p* < 0.05 was considered statistically significant.

## 3 Results

### 3.1 Analysis of Baseline Characteristics

This study enrolled 104 elderly patients (≥ 65 years) with unresectable stage III NSCLC to receive induction chemoimmunotherapy. As shown in Table 1, the distribution of clinicopathological characteristics—including gender, age, smoking status, ECOG score, pathological type, clinical stage, treatment regimen, and radiotherapy dose—in the entire cohort was not statistically different between subgroups stratified by MLD (< 12 Gy vs. ≥ 12 Gy), MHD (< 10 Gy vs. ≥ 10 Gy), MBD (< 6 Gy vs. ≥ 6 Gy), and EDRIC (< 6.4 Gy vs. ≥ 6.4 Gy) (all *p* > 0.05). This balanced baseline distribution minimizes the potential influence of confounding variables on subsequent predictive analyses.

**TABLE 1:** Baseline demographic and clinical characteristics of the patients.

Characteristics	MLD		*p*	MHD		*p*	MBD		*p*	EDRIC		*p*
	< 12	≥ 12		< 10	≥ 10		< 6	≥ 6		< 6.4	≥ 6.4	
Sex												
Male	47	48	0.564	45	50	0.647	57	38	0.232	50	45	0.448
Female	3	6		3	6		3	6		3	6	
Age												
< 70	28	29	0.814	27	30	0.784	33	24	0.963	29	28	0.985
≥ 70	22	25		21	26		27	20		24	23	
Smoking												
Never	6	14	0.072	8	12	0.539	11	9	0.786	9	11	0.553
Former/current	44	40		40	44		49	35		44	40	
ECOG												
0	1	4	0.122	2	3	0.401	2	3	0.501	2	3	0.442
1	35	43		34	44		46	32		38	40	
2	12	7		10	9		10	9		11	8	
3	2	0		2	0		2	0		2	0	
WHO histology												
Squamous	42	38	0.197	38	42	0.824	48	32	0.371	41	39	0.546
Non‐squamous	6	14		8	12		11	9		9	11	
NOS	2	2		2	2		1	3		3	1	
Stage												
IIIA	16	18	0.482	13	21	0.089	23	11	0.193	20	14	0.151
IIIB	27	24		29	22		29	22		27	24	
IIIC	7	12		6	13		8	11		6	13	
CRT modality												
Sequential	42	45	0.927	39	48	0.539	53	34	0.132	41	46	0.111
Concurrent	8	9		9	8		7	10		12	5	
Dose (Gy)												
≥ 54	41	47	0.477	42	46	0.450	50	38	0.672	45	43	1.000
< 54	9	7		6	10		10	6		8	8	

Abbreviations: CRT: chemoradiotherapy; EDRIC: estimated dose of radiation to immune cells; MBD: mean body dose; MHD: mean heart dose; MLD: mean lung dose; NOS: not otherwise specified.

### 3.2 Survival Analysis Results

For the entire cohort, the PFS for the entire cohort was 23.9 months, with 1‐ and 2‐year PFS rates of 78.2% and 49.1%, respectively. The median OS was 46 months, accompanied by 1‐ and 2‐year OS rates of 90.9% and 71.8%, respectively.

#### 3.2.1 Progression‐Free Survival

##### 3.2.1.1 Differences in Survival Curves

**FIGURE 1:**
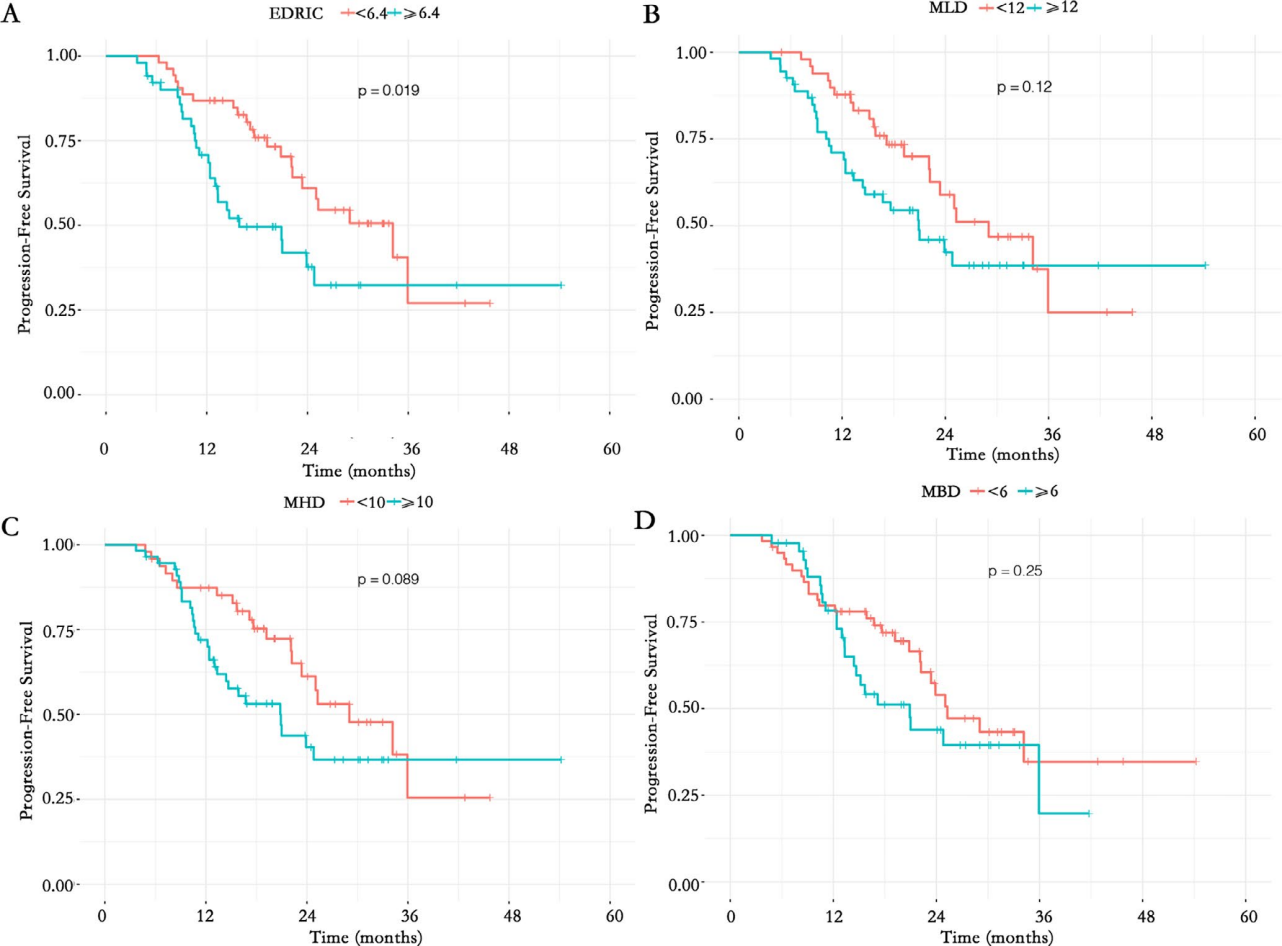
Kaplan–Meier curves of PFS for (A) EDRIC, (B) MLD, (C) MHD, and (D) MBD.

Kaplan–Meier survival analysis (Figure 1) showed that the PFS in the EDRIC ≥ 6.4 Gy group was significantly shorter than that in the EDRIC < 6.4 Gy group (*p* = 0.019); in contrast, there were no statistically significant differences in PFS between subgroups stratified by MLD (*p* = 0.12), MHD (*p* = 0.089), or MBD (*p* = 0.25).

##### 3.2.1.2 Univariate and Multivariate Analyses

The results of univariate Cox regression analysis (Table 2) showed that only EDRIC ≥ 6.4 Gy was associated with shorter PFS (HR = 1.951, *p* = 0.019), while MLD, MHD, and MBD were not significantly associated with PFS (all *p* > 0.05).

**TABLE 2:** Univariate and multivariate analyses of PFS in the patients.

Characteristics	Univariate analysis		Multivariate analysis	
	HR (95% CI)	*p*	HR (95% CI)	*p*
Sex				
Male	Ref			
Female	0.587 (0.182–1.893)	0.373		
Age				
< 70	Ref			
≥ 70	1.433 (0.817–2.516)	0.210		
Smoking				
Never	Ref			
Former/current	1.009 (0.489–2.080)	0.981		
ECOG				
0	Ref			
1	1.817 (0.300–11.008)	0.516		
2	0.670 (0.160–2.805)	0.583		
3	0.922 (0.194–4.393)	0.919		
WHO histology				
Squamous	Ref			
Non‐squamous	2.177 (0.299–15.862)	0.443		
NOS	1.282 (0.159–10.306)	0.815		
Stage				
IIIA	Ref			
IIIB	0.604 (0.277–1.319)	0.206		
IIIC	0.617 (0.300–1.270)	0.190		
CRT modality				
Sequential	Ref			
Concurrent	0.764 (0.342–1.708)	0.512		
Dose (Gy)				
≥ 54	Ref			
< 54	1.466 (0.675–3.182)	0.334		
MLD				
< 12	Ref			
≥ 12	1.560 (0.884–2.753)	0.120		
MHD			1.240 (0.640–2.400)	0.524
< 10	Ref			
≥ 10	1.636 (0.922–2.902)	0.089		
MBD				
< 6	Ref			
≥ 6	1.389 (0.791–2.439)	0.250		
EDRIC			1.852 (1.009–3.478)	0.049
< 6.4	Ref			
≥ 6.4	1.951 (1.104–3.448)	0.019		

Abbreviations: CRT: chemoradiotherapy; EDRIC: estimated dose of radiation to immune cells; MBD: mean body dose; MHD: mean heart dose; MLD: mean lung dose; NOS: not otherwise specified; PFS: progression‐free survival.

In multivariate analysis, EDRIC ≥ 6.4 Gy remained an independent predictor of worse PFS (HR = 1.852, 95% CI: 1.009–3.478, *p* = 0.049), while conventional dosimetric parameters lacked prognostic significance.

#### 3.2.2 Overall Survival

##### 3.2.2.1 Differences in Survival Curves

**FIGURE 2:**
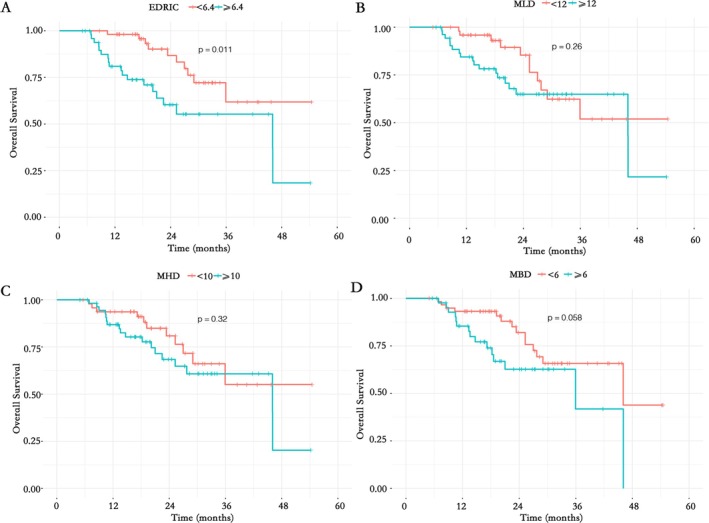
Kaplan–Meier curves of OS for (A) EDRIC, (B) MLD, (C) MHD, and (D) MBD.

Kaplan–Meier curves (Figure 2) demonstrated markedly shorter OS in the EDRIC ≥ 6.4 Gy group (*p* = 0.011). No significant differences were observed for MLD (*p* = 0.26), MHD (*p* = 0.32), or MBD (*p* = 0.058).

##### 3.2.2.2 Univariate and Multivariate Analyses

Univariate Cox analysis (Table 3) showed that EDRIC ≥ 6.4 Gy (HR = 2.650, 95% CI: 1.219–5.761, *p* = 0.011) and age ≥ 70 years (HR = 2.207, 95% CI: 1.024–4.756, *p* = 0.043) were associated with shortened OS; MLD, MHD, and MBD were not significantly associated with OS (all *p* > 0.05). Multivariate analysis further verified that both EDRIC ≥ 6.4 Gy (HR = 2.289, 95% CI: 0.989–5.488, *p* = 0.048) and age ≥ 70 years (HR = 2.870, 95% CI: 1.279–6.444, *p* = 0.011) as independent risk factors for OS.

**TABLE 3:** Univariate and multivariate analyses of OS in the patients.

Characteristics	Univariate analysis		Multivariate analysis	
	HR (95% CI)	*p*	HR (95% CI)	*p*
Sex				
Male	Ref			
Female	0.825 (0.195–3.494)	0.794		
Age			2.870 (1.279–6.444)	0.011
< 70	Ref			
≥ 70	2.207 (1.024–4.756)	0.043		
Smoking				
Never	Ref			
Former/current	0.841 (0.341–2.075)	0.707		
ECOG				
0	Ref			
1	0.935 (0.130–6.738)	0.946		
2	0.346 (0.079–1.508)	0.158		
3	0.479 (0.089–2.581)	0.391		
WHO histology				
Squamous	Ref			
Non‐squamous	NS	0.950		
NOS	NS	0.954		
Stage				
IIIA	Ref			
IIIB	0.793 (0.290–2.172)	0.652		
IIIC	0.617 (0.229–1.659)	0.338		
CRT modality				
Sequential	Ref			
Concurrent	0.396 (0.094–1.677)	0.209		
Dose (Gy)			3.550 (1.405–8.970)	0.007
≥ 54	Ref			
< 54	3.072 (1.258–7.501)	0.014		
MLD				
< 12	Ref			
≥ 12	1.542 (0.726–3.278)	0.260		
MHD				
< 10	Ref			
≥ 10	1.464 (0.689–3.111)	0.320		
MBD			1.359 (0.582–3.173)	0.478
< 6	Ref			
≥ 6	2.006 (0.963–4.180)	0.058		
EDRIC			2.289 (0.989–5.488)	0.048
< 6.4	Ref			
≥ 6.4	2.650 (1.219–5.761)	0.011		

Abbreviations: CRT: chemoradiotherapy; EDRIC: estimated dose of radiation to immune cells; MBD: mean body dose; MHD: mean heart dose; MLD: mean lung dose; NOS: not otherwise specified; OS: overall survival.

### 3.3 Comparison of Predictive Efficacy

#### 3.3.1 Predictive Accuracy for PFS

**FIGURE 3:**
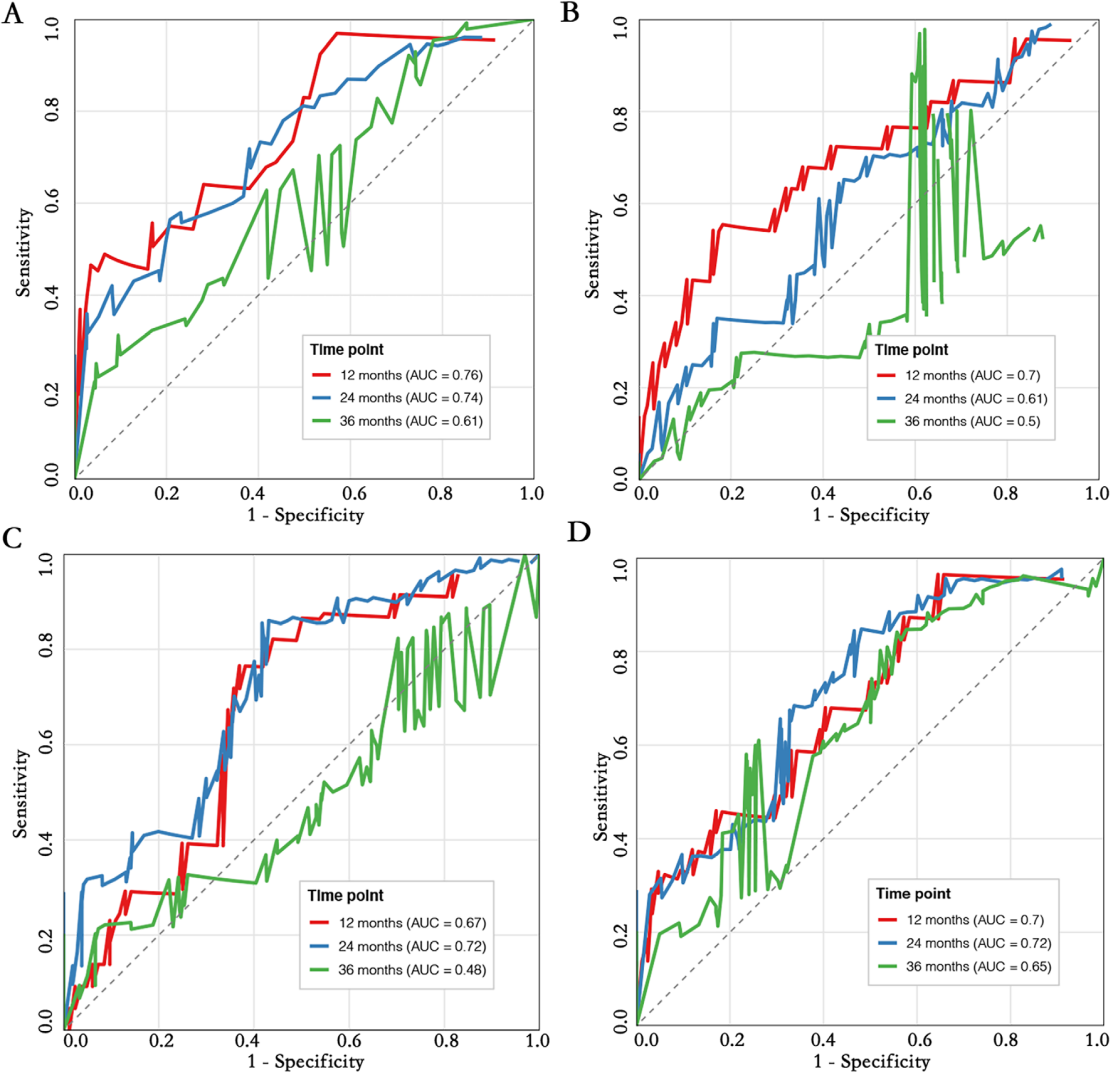
ROC analysis of PFS at 1‐, 2‐, and 3‐year intervals in (A) EDRIC, (B) MLD, (C) MHD, and (D) MBD.

Time‐dependent ROC curve analysis for 1‐, 2‐, and 3‐year PFS showed that the predictive efficacy of EDRIC was superior to that of MLD, MHD, and MBD. Specifically, the AUC of EDRIC at 12 months was 0.76, higher than that of MLD (0.70), MHD (0.67), and MBD (0.7); at 24 months, the AUC of EDRIC was 0.74, higher than that of MLD (0.61), MHD (0.72), and MBD (0.72); at 36 months, the AUC of EDRIC (0.61) was higher than that of MLD (0.5) and MHD (0.43) (Figure 3).

#### 3.3.2 Predictive Accuracy for OS

**FIGURE 4:**
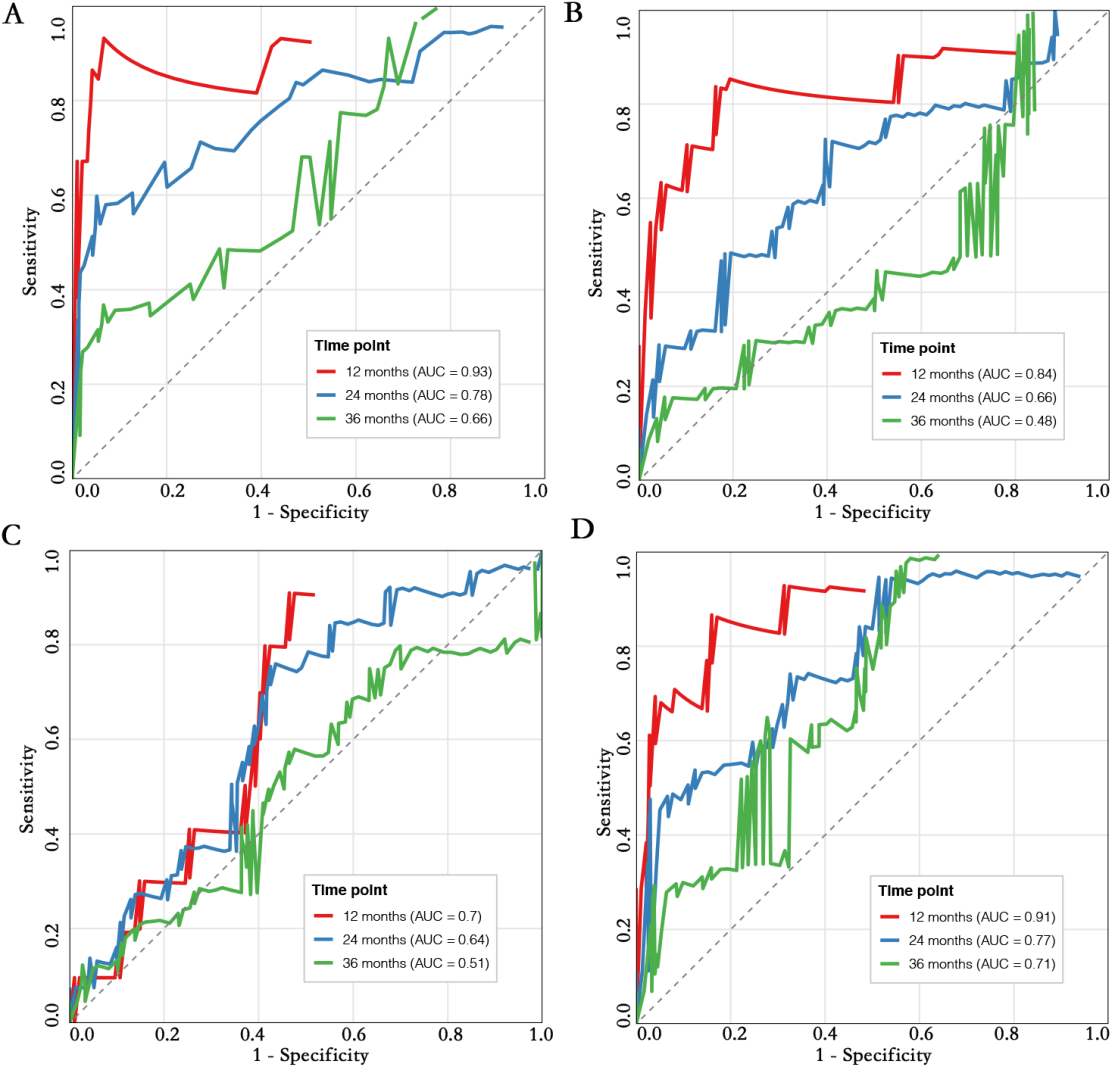
ROC analysis of OS at 1‐, 2‐, and 3‐year intervals in (A) EDRIC, (B) MLD, (C) MHD, and (D) MBD.

For the prediction of OS, EDRIC also showed excellent predictive accuracy. Among them, the AUC of EDRIC at 12 months was 0.93, higher than that of MLD (0.84), MHD (0.7), and MBD (0.91); at 24 months, the AUC of EDRIC was 0.78, higher than that of MLD (0.66), MHD (0.64), and MBD (0.77); at 36 months, the AUC of EDRIC (0.66) was higher than that of MLD (0.48) and MHD (0.51) (Figure 4).

## 4 Discussion

This two‐center real‐world study systematically explored the predictive value of the novel dosimetric parameter EDRIC for radiotherapy outcomes in elderly patients with stage III unresectable NSCLC after Induction chemoimmunotherapy for the first time, and compared it with traditional dosimetric parameters (MLD, MHD, MBD). Based on clinical data analysis, survival curves, and ROC comparisons, this study confirmed that EDRIC shows significant potential in predicting survival outcomes and guiding individualized radiotherapy for this population.

This study verified the predictive value of EDRIC for radiotherapy outcomes in elderly patients with stage III unresectable NSCLC: the results showed that EDRIC ≥ 6.4 Gy was an independent risk factor for shortened PFS and OS in patients (PFS: HR = 1.852, *p* = 0.049; OS: HR = 2.289, *p* = 0.048), with significant differences in survival curves between groups (PFS: *p* = 0.019; OS: *p* = 0.011). It is speculated that the mechanism is that higher radiation dose to immune cells leads to immunosuppression, thereby reducing the body's ability to control tumor growth and metastasis and the effect of immunotherapy [13]. This verification result is consistent with the conclusions of previous similar studies, collectively confirming the cross‐population predictive efficacy of EDRIC in radiotherapy for elderly NSCLC, and laying a solid evidence‐based foundation for the breadth of its clinical application [16]. It also establishes the rationality and necessity for the subsequent horizontal comparison of its advantages and disadvantages with MLD, MHD and MBD.

The EDRIC cutoff of 6.4 Gy was determined as the cohort median, an objective method aligned with foundational studies in this field [13]. While this cohort‐specific value successfully identified subgroups with distinct survival outcomes in our analysis, its variation from other reported medians emphasizes that our result is a robust proof‐of‐concept. The definitive clinical threshold for EDRIC must be established through future prospective, multi‐center validation.

MLD, MHD and MBD did not show stable predictive value in this study: there were no statistically significant differences in PFS and OS between high and low groups (all *p* > 0.05), and they were not independent risk factors in the multivariate model. This is closely related to the design limitations of traditional parameters. First, traditional indicators have the limitation of single‐organ focus: MLD mainly reflects radiation damage to lung tissue, but its association with radiation pneumonitis did not affect the survival of patients in this study; although MHD is associated with myocardial damage, the main cause of declining cardiac reserve function in the elderly is arteriosclerosis, which has a weak association with acute radiation damage [17]; MBD involves whole‐body dose but cannot distinguish the radiation sensitivity between bone marrow hematopoietic areas and muscle tissue, making it difficult to capture damage to immune hubs such as the thymus and lymph nodes [18]. Second, traditional indicators cannot integrate immune‐tumor interactions: traditional parameters do not include immune organ weights, while the prognosis of elderly patients is highly dependent on the impact of radiotherapy on immune function [19, 20, 21]. For example, although the MBD ≥ 6 Gy group showed a trend of shortened OS (*p* = 0.058), it could not reflect the degree of immunosuppression because it did not quantify the association between bone marrow hematopoietic stem cell damage and dendritic cell dysfunction, so its efficacy was weaker than that of EDRIC.

EDRIC has multiple advantages: first, it has stronger predictive accuracy, with higher AUC values for PFS and OS than traditional parameters, enabling better customization of treatment plans for elderly patients; second, it can integrate multiple parameters such as MLD, MHD, MBD, and irradiation frequency; third, in comparison, the composite design of EDRIC (integrating MLD, MHD, MBD, and number of fractions) provides a comprehensive assessment consistent with the complex pathophysiology of aging and cancer progression, and is more in line with the pathophysiological characteristics of “immune senescence + tumor progression” in elderly patients [13, 19]. ROC curve confirmed that the AUC values of EDRIC for 1–3‐year PFS and OS were higher than those of traditional parameters, especially the AUC for 12‐month OS reached 0.93, which was significantly better than MLD (0.84) and MBD (0.70). The advantages of EDRIC support its role as a core indicator for optimizing radiotherapy plans in elderly patients, helping to balance local control and immune protection, and can optimize tumor control effects while reducing damage to the immune system [12]. This is particularly important for elderly patients with age‐related decline in immunity, as it can better balance local control and immune protection compared to traditional parameters [22, 23].

In addition, it is worth noting that old age is an independent risk factor for shortened OS, which, like other similar studies, indicates that further advanced age in elderly patients may exacerbate the risk of poor prognosis [22, 24, 25]. Mechanistically, this may be related to the further decline in immune function with age in this population and the increased infiltration of immunosuppressive cells in the tumor microenvironment. At the same time, older individuals often have more declined organ function reserves and a higher proportion of comorbidities, which may not only limit the application of radical treatment regimens but also accelerate tumor progression and the occurrence of treatment‐related adverse reactions [24, 25, 26, 27, 28, 29]. Our finding that age ≥ 70 years was an independent predictor for OS underscores the heterogeneity within the geriatric population. To better personalize treatment for the most vulnerable patients, future studies should aim to include and stratify by more granular age subgroups (65–74, 75–84, ≥ 85 years) to refine predictive models, particularly for the “oldest old”. In this case, EDRIC, as a parameter integrating the dose to immune organs, is more likely to be more suitable as a reference for selecting radiotherapy dose and regimens compared to traditional indicators.

This study has several limitations that should be acknowledged. First, the direct relationship between EDRIC and immune function indicators has yet to be validated through laboratory experiments. Second, the subgroup analysis of patients aged ≥ 70 years was limited by the small sample size, and the predictive efficacy in elderly populations—particularly those aged ≥ 80 years—requires further verification in larger cohort studies. Third, the high proportion of squamous cell carcinoma (77%) and the predominant use of sequential chemoradiotherapy (83%) in our cohort may limit the generalizability of our findings to populations with different histologic distributions or where concurrent chemoradiotherapy is the standard. These factors reflect the real‐world treatment patterns in the participating centers for elderly patients, who often present with comorbidities precluding concurrent therapy, but they underscore the need for validation in more diverse cohorts. Fourth, EDRIC may be influenced by tumor volume and location, as larger PTVs could inherently encompass more lymphoid tissue, potentially confounding the association between EDRIC and survival. A future analysis matching patients by PTV volume to compare dosimetry and EDRIC would be highly valuable to isolate the effect of radiation technique and dose on immune cell exposure. These limitations underscore important directions for future prospective research.

## 5 Conclusion

This multicenter real‐world study demonstrates that EDRIC shows potential in predicting PFS and OS for elderly stage III unresectable NSCLC patients, with 6.4 Gy as a potential threshold for optimizing radiotherapy by balancing local control and immune protection. Our findings support the hypothesis that EDRIC may be a valuable dosimetric parameter and warrant further investigation in prospective studies to validate its clinical utility.

## Author Contributions

**Huan Li:** conceptualization, writing – review and editing, writing – original draft. **Xingyu Du:** data curation, software. **Song Guan:** methodology, software. **Hui Wang and Yan Xing:** data curation. **Cuimeng Tian:** validation, visualization. **Li Wen:** writing – review and editing, supervision, visualization, formal analysis.

## Conflicts of Interest

The authors declare no conflicts of interest.

## Data Availability

The data that support the findings of this study are available from the corresponding author upon reasonable request.
